# Doctor Referral of Overweight People to Low Energy total diet replacement Treatment (DROPLET): pragmatic randomised controlled trial

**DOI:** 10.1136/bmj.k3760

**Published:** 2018-09-26

**Authors:** Nerys M Astbury, Paul Aveyard, Alecia Nickless, Kathryn Hood, Kate Corfield, Rebecca Lowe, Susan A Jebb

**Affiliations:** Nuffield Department of Primary Care Health Sciences, University of Oxford, UK

## Abstract

**Objective:**

To test the effectiveness and safety of a total diet replacement (TDR) programme for routine treatment of obesity in a primary care setting.

**Design:**

Pragmatic, two arm, parallel group, open label, individually randomised controlled trial.

**Setting:**

10 primary care practices in Oxfordshire, UK.

**Participants:**

278 adults who were obese and seeking support to lose weight: 138 were assigned to the TDR programme and 140 to usual care. 73% of participants were re-measured at 12 months.

**Interventions:**

The TDR programme comprised weekly behavioural support for 12 weeks and monthly support for three months, with formula food products providing 810 kcal/day (3389 kJ/day) as the sole food during the first eight weeks followed by reintroduction of food. Usual care comprised behavioural support for weight loss from a practice nurse and a diet programme with modest energy restriction.

**Main outcome measures:**

The primary outcome was weight change at 12 months analysed as intention to treat with mixed effects models. Secondary outcomes included biomarkers of cardiovascular and metabolic risk. Adverse events were recorded.

**Results:**

Participants in the TDR group lost more weight (−10.7 kg) than those in the usual care group (−3.1 kg): adjusted mean difference −7.2 kg (95% confidence interval −9.4 to −4.9 kg). 45% of participants in the TDR group and 15% in the usual care group experienced weight losses of 10% or more. The TDR group showed greater improvements in biomarkers of cardiovascular and metabolic risk than the usual care group. 11% of participants in the TDR group and 12% in the usual care group experienced adverse events of moderate or greater severity.

**Conclusions:**

Compared with regular weight loss support from a practice nurse, a programme of weekly behavioural support and total diet replacement providing 810 kcal/day seems to be tolerable, and leads to substantially greater weight loss and greater improvements in the risk of cardiometabolic disease.

**Trial registration:**

International Standard Randomised Controlled Trials No ISRCTN75092026.

**Figure:**
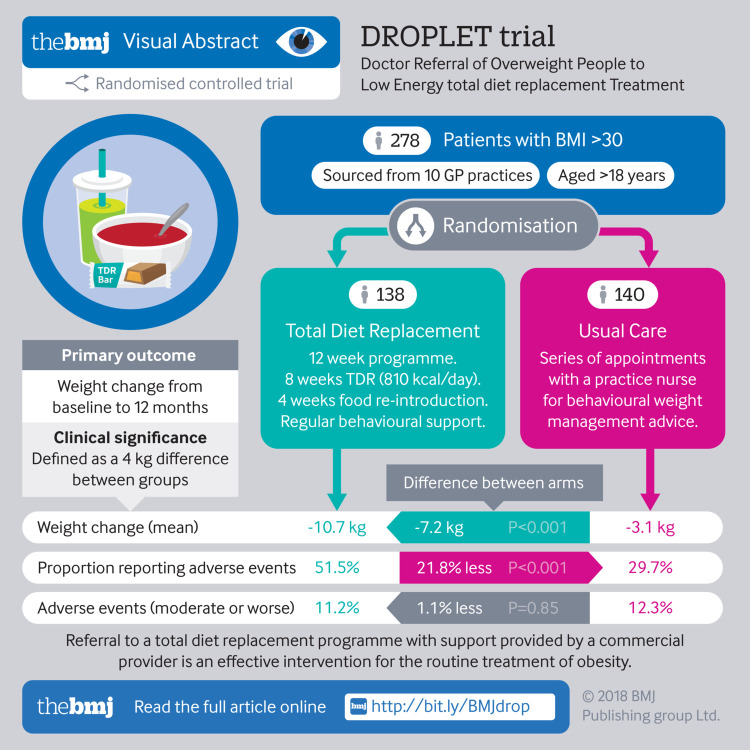


## Introduction

Excess adiposity is one of the main risk factors for morbidity and mortality.^1^ Weight loss ameliorates the risk and improves functioning and wellbeing,^2^ with growing evidence that benefits can persist even if weight is regained.^2^ ^3^ Primary care doctors have the opportunity to offer treatments for obesity at the scale required to have a discernible effect on prevalence of obesity and related diseases,^4^ and they are encouraged to screen patients and offer support for weight loss.^5^ Despite this, doctors rarely provide such support.^6^

Good evidence shows that weight loss programmes provided in community groups by commercial providers are more effective than routine management delivered by primary care clinicians.^7^ ^8^ ^9^ Moreover, such programmes are cost effective and, over the long term, cost saving.^8^ Notwithstanding the population benefits, those who are referred lose only an additional 2 kg compared with self help interventions, and people would benefit from interventions that lead to greater weight loss.^10^ One option is a total diet replacement (TDR) programme, combining a low energy diet with behavioural support. In a systematic review of trials we found that very low energy diets providing <800 kcal/day led to statistically significantly greater weight loss than behavioural weight management programmes based on usual foods.^11^ Currently, TDR programmes offer up to 1200 kcal/day, but similarly use formula food products as the sole source of nutrition alongside a behavioural support programme. Most trials of these programmes have been small scale, and conducted in research settings or specialist obesity clinics. A common but unsupported perception is that such programmes are unacceptable to most people, possibly unsafe, and lead to rapid weight regain, and guidelines do not recommend their use for general treatment of obesity.^12^ ^13^

We investigated the effectiveness and safety of referral by a primary care doctor to a commercially provided low energy TDR programme compared with usual care. Consistent with the pragmatic nature of the trial we did not attempt to match treatment intensity in the two groups; rather, we compared the Cambridge Weight Plan programme, comprising specially formulated products and behavioural support, with the usual type of weight management programmes offered by primary care staff, based on dietary advice and behavioural support.

## Methods

This trial was a pragmatic, individually randomised, two arm, open label, parallel design with a practice nurse allocating participants to a TDR programme or routine support.^14^ Participants gave written informed consent. On the advice of the independent trial steering committee, the protocol was amended after registration to reduce the number of secondary outcomes.

### Participants and setting

We recruited participants from primary care practices in Oxfordshire, UK that were willing and able to offer a weight management programme within the practice. General practitioners searched their electronic health records for adults with a body mass index (BMI) of at least 30 kg/m^2^ and whose health would benefit from weight loss and invited them by letter to participate. We excluded people who had received or were scheduled for bariatric surgery, those participating in a weight management programme, or those with contraindications to the TDR according to the protocol.^14^ (The supplementary appendix presents the full inclusion and exclusion criteria.) After telephone screening by researchers, eligible participants scheduled an appointment with a nurse at their local practice.

### Randomisation and masking

An independent statistician produced a computer generated randomisation list with 1:1 allocation using stratified block randomisation with randomly permuted block sizes of 2, 4, and 6, stratified by general practice and BMI (≤35 or >35). After the nurse had confirmed eligibility, participants were enrolled in the study and the allocation was revealed using an online randomisation programme to ensure full allocation concealment. Owing to the type of intervention it was not possible to blind participants, clinicians, or some of the researchers to treatment allocation.

### Interventions

The TDR programme was provided by Cambridge Weight Plan UK, which manages a network of counsellors providing behavioural support and food products.^14^ Participants were asked to contact a local counsellor who was aware of the research study and the protocol for the provision of formula food products, but who had not received any additional training to deliver the behavioural support programme. For the first 12 weeks, participants met with the counsellor weekly for support, which comprised goal setting, feedback, encouragement, reassurance, and problem solving. Participants replaced all food with four formula food products daily (soups, shakes, and bars), 750 mL of skimmed milk, 2.25 L of water or other low or no energy drinks, and a fibre supplement; energy intake comprised 810 kcal/day (3389 kJ/day) (see supplementary table S1). After eight weeks, there was a four week stepwise reduction in use of the formula food products and reintroduction of conventional food based meals. During the weight maintenance phase from week 13 to 24, counsellors encouraged participants to attend monthly appointments and to consume one formula food product a day, with the remainder of the diet provided by food. If weight was regained, the protocol allowed for participants to return to the TDR stage for up to four weeks. This programme was free of charge to week 24.

We asked clinicians to review drugs for any participants randomised to TDR who were receiving treatment for type 2 diabetes, or hypertension, or taking fibrates at the start of the programme, and at a scheduled assessment at one month and in routine reviews or as needed thereafter. We supplied clinicians with guidelines for this (see supplementary appendix). Data on the changes made to participants’ drugs were collected at follow-up visits, but changes to drugs were not one of the prespecified outcomes in the protocol. We plan on reporting this as a secondary analysis elsewhere.

For the comparator, participants followed each practice’s usual weight management protocol.^14^ We asked nurses to offer a programme for 12 weeks, at a frequency usual to the practice (eg, weekly or biweekly). Participants also received a 47 page booklet “So you want to lose weight . . . for good,”^15^ which includes information on goal setting, monitoring, and feedback, and advice about food types, portion control, and physical activity.

Participants were not prevented from attending other weight management groups, but no National Health Service referrals to these schemes were offered during the intervention period.

### Procedures

We measured height at baseline only, blood samples at baseline and 12 months, and all other measurements at baseline and 3, 6, and 12 months. A digital scale (TANITA SC-240; Tanita, Amsterdam, Netherlands) was used to measure weight and body fat. Waist circumference was measured at the top of the iliac crest, and blood pressure was measured in triplicate using an automated blood pressure monitor and with participants seated, with the mean of the last two readings recorded. We recorded quality of life using two instruments: the EQ-5D and obesity and weight loss quality of life (OWL-QOL).^16^ ^17^ Fasting blood samples were collected to measure blood glucose, insulin, and triglyceride levels and cholesterol fractions. Practice staff took the measurements at baseline and the research team at 3, 6, and 12 months. At 12 months we asked participants to self report if they had continued to try to manage their weight, and the methods they had used.

### Outcomes

The primary outcome was change in body weight from baseline to 12 months. Prespecified secondary outcomes were change in body weight between baseline and three and six months, the proportion of participants achieving 5% or more and 10% or more weight loss at 12 months, and change in fat mass, low density lipoprotein cholesterol level, glycated haemoglobin (HbA_1c_), and systolic and diastolic blood pressure at 12 months.

Prespecified exploratory outcomes were change in fat mass and blood pressure at three and six months and in waist circumference at 3, 6, and 12 months. Change in concentrations of fasting triglycerides, high density lipoprotein cholesterol, glucose, and insulin from baseline to 12 months was measured. The HOMA (Homeostatic model assessment) model was used to measure insulin resistance (HOMA-IR), β cell function (HOMA-β), and insulin sensitivity (HOMA-S) and QRISK2 to calculate the change in 10 year cardiovascular risk.^18^ ^19^ We measured the change in self reported quality of life between baseline and 12 months using the EQ-5D and OWL-QOL.^16^ ^17^

The research team recorded adverse events through semistructured, open ended questioning of participants, by phone or in person, during the first three months of the programme. To allow for diagnostic delay of gallstone related events, we asked specific questions about such events at six months. These were coded using MedDRA version 18.1 and presented at the system organ class and preferred term level. In accordance with the statistical analysis plan, we present events that occurred in at least 2% of the participants, and all serious adverse events.

### Statistical analysis

We determined a difference of 4 kg weight loss between groups to be clinically relevant. Using data on standard deviation from published studies and assuming 90% power with a two sided significance of 5%, and 20% loss to follow-up, we needed a sample of 270 people. Accounting for multiple testing of secondary outcomes, this gave 90% power to detect a standardised difference of 0.56 with 5% significance for the secondary outcomes.

We followed a statistical analysis plan approved by the independent trial steering committee before database lock. An independent trial statistician used PROC MIXED in SAS Version 9.4 to analyse the primary, secondary, and exploratory outcomes using an intention to treat analysis. We used linear mixed effects models with an unstructured correlation matrix for repeated measures and adjusted for baseline stratification variables, with practice set as a random effect. For binary outcomes, we used analogous logistic models. Before analysis of outcomes, we assessed the association between baseline variables and loss to follow-up at 12 months. Age and sex were associated with loss to follow-up and so were included as covariates as planned.

We assessed the sensitivity of the results to missing data using different imputation methods; baseline and last observation carried forward, completers only, multiple imputation, and a pattern mixture model assuming different degrees of missing not at random. To assess whether treatment effects differed by age, sex, BMI, socioeconomic status (based on participant’s postcode) and practice, we performed prespecified exploratory subgroup analyses. Following our statistical plan, we did not compare change in quality of life using inferential statistics.

### Patient and public involvement

Our extensive public involvement activities have shown that a large proportion of people are interested to know whether weight loss interventions are effective, and they welcome this kind of research. In a previous trial involving opportunistic offers of support for weight loss, patients overwhelmingly reported that this was appropriate and helpful.^5^ Members of the public who have expressed an interest in our research were invited to comment on the design of the study and the patient-facing materials before ethical submission. Two lay people were members of the trial steering committee. Participants in the trial were offered the opportunity to hear the results of the study upon completion, and a lay summary and infographic have been provided.

## Results

Participants were recruited between 12 January 2016 and 28 July 2016. Of 286 participants screened from 10 practices, 278 were eligible and randomly allocated to either a total diet replacement (TDR) programme (n=138) or usual care (n=140). Follow-up was completed on 4 August 2017.

**Fig 1:**
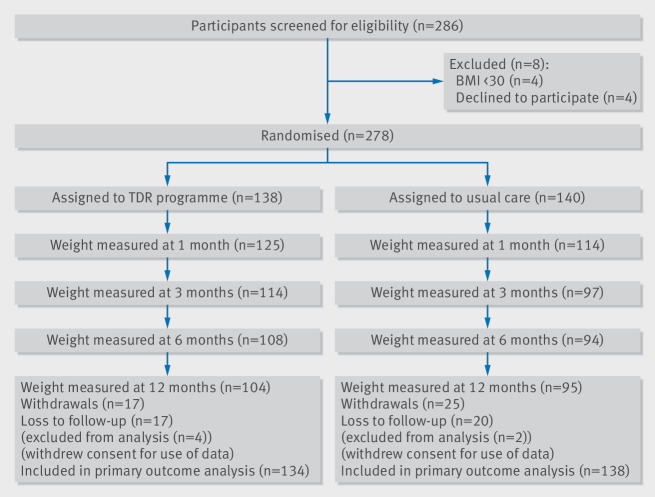
Consort flowchart. BMI=body mass index; TDR=total diet replacement

The average age of participants was 48 (SD 12) years, 61% were women, and 88% were white British. The average BMI was 37.2 (SD 5.4). On enrolment 23% had a diagnosis of hypertension and 15% had diabetes (table 1). Overall, 138 participants were randomised to the TDR group and 140 to usual care. After randomisation, four and two participants, respectively, withdrew consent for their data to be used. At 12 months we followed-up 104 (78%) participants in the TDR group and 95 (69%) in the usual care group (fig 1).

**Table 1:** Baseline characteristics of participants assigned to a total replacement diet (TDR) programme or usual care. Values are means (standard deviations) unless stated otherwise

Characteristics	Usual care group (n=140)*	TDR group (n=138)*
Age (years)	47.4 (12.8)	48.2 (11.5)
Sex:		
No (%) women	84 (60)	81 (60.5)
No (%) men	54 (39)	53 (40)
Ethnicity:		
No (%) white British	119 (86)	121 (90)
No (%) not white British	19 (14)	13 (10)
Index of multiple deprivation 10th†	7.3 (2.0)	7.6 (2.0)
Weight (kg)	105.2 (20)	107.9 (18.9)
Height (cm)	168.7 (9.7)	169.2 (9.5)
Body mass index	36.8 (5.1)	37.6 (5.7)
Waist circumference (cm)	115.0 (12.5)	116.4 (13.5)
Body fat (%)	42.1 (7.7)	43.0 (7.8)
Blood pressure (mm Hg):		
Systolic	130.1 (15.8)	130.6 (16.4)
Diastolic	81.3 (9.9)	83.1 (9.7)
No (%) with medical condition:		
Type 2 diabetes	20 (14)	21 (16)
Hypertension	30 (22)	33 (25)
HbA_1c_ (mmol/mol)	38.6 (10.9)	39.6 (12.4)
Fasting blood glucose (mmol/L)	5.6 (1.9)	5.9 (2.4)
Fasting insulin (pmol/L)	105.3 (85.7)	96.4 (48.1)
Cholesterol (mmol/L):		
Total	5.1 (1.1)	5.1 (1.1)
High density lipoprotein	1.2 (0.4)	1.2 (0.3)
Low density lipoprotein	3.2 (0.9)	3.2 (0.9)
Triglycerides (mmol/L)	1.6 (0.9)	1.6 (0.8)

* Two people in the usual care group and four in the TDR group withdrew consent for use of data after randomisation.

† Indicator of deprivation, with first 10th being most deprived and fifth 10th least deprived.

In addition to the intervention set out in the protocol, during the weight maintenance phase (12-24 weeks) 12 participants (9%) in the TDR group chose to pay for additional products or support. At the 12 month follow-up, 104 participants in the TDR group and 95 in the usual care group provided information about ongoing weight control practices. Of these, 71 (68%) in the TDR group reported trying to lose weight, 27 (26%) were in contact with the TDR provider, and four were attending a community weight loss group. In the usual care group, 73 (77%) reported trying to lose weight, four were continuing to follow the plan recommended by the nurse, four were in contact with a TDR provider, and eight were attending a community weight loss group.

### Primary outcome

**Fig 2:**
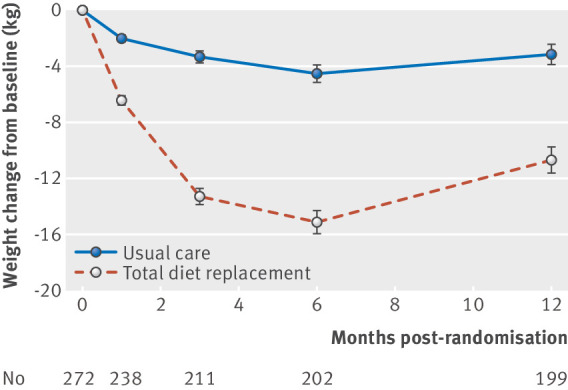
Weight change over 12 months in intention to treat population. Values represent mean (standard error of the mean)

Mean weight change at 12 months was −10.7 (SD 9.6) kg in the TDR group and −3.1 (7.0) kg in the usual care group (fig 2). The adjusted difference in mean weight change between the TDR and control groups was −7.2 kg (95% confidence interval −9.4 to −4.9 kg; P<0.001).

Sensitivity analyses on loss to follow-up did not change the conclusion that the TDR programme led to greater weight change than usual care (see supplementary table S2). In the pattern mixture modelling (see supplementary fig S1), even assuming extreme bias in loss to follow-up in the TDR group or usual care group, there was a treatment difference of more than 5 kg in favour of TDR. Furthermore, there was no evidence that the intervention effect on weight differed by sex (P=0.22), age group (P=0.85), socioeconomic status (P=0.65), BMI (P=0.09), diabetes status (P=0.42), or practice (P=0.22) (see supplementary fig S2).

### Secondary outcomes

The TDR group had greater weight change at all time points before 12 months. At three months, the adjusted difference between groups was −9.6 kg (95% confidence interval −11.0 to −8.2 kg; P<0.001) and at six months was −9.6 kg (−11.6 to −7.7 kg; P<0.001).

Prespecified secondary outcomes included change in fat mass (measured at same time as body weight), which showed similar, albeit slightly smaller, differences in favour of the TDR group. Of those participants followed up at 12 months, 73% in the TDR group (n=104) and 32% in the usual care group (n=95) had lost 5% or more of their baseline body weight (adjusted odds ratio 6.5, 95% confidence interval 3.4 to 12.2; P<0. 001), and 45% and 15%, respectively, had lost 10% or more of their baseline body weight (4.9, 2.4 to 9.9; P<0.001) (see supplementary fig S2). The number needed to treat to observe these benefits was 2.4 (95% confidence interval 1.8 to 3.5) and 3.3 (2.4 to 5.4) for 5% or more and 10% or more body weight loss, respectively. In an addition to the protocol and in response to peer review, we report that 22% of participants in the TDR group and 4% in the usual care group lost 15% or more of their body weight at 12 month follow-up.

At 12 months, participants in the TDR group had greater reductions in HbA_1c_ (adjusted difference −2.2 mmol/mol, 95% confidence interval −4.4 to 0.0 mmol/mol; P=0.05) and diastolic blood pressure (−3.1 mm Hg, −5.5 to −0.7 mm Hg; P= 0.01), but no significant statistical difference was found in the reduction in systolic blood pressure (−2.9 mm Hg, −6.4 to 0.6 mm Hg; P=0.1) or low density lipoprotein cholesterol level (0.0 mmol/L, 95% confidence interval −0.2 to 0.2 mmol/L; P=0.8). Supplementary table S6 shows the outcomes for patients with a diagnosis of type 2 diabetes at baseline.

### Exploratory outcomes

The change in waist circumference showed a similar pattern to that of changes in body weight and fat mass. Other exploratory outcomes assessed changes in glucose regulation, blood pressure, and other lipid fractions. These also reflected the secondary outcomes, showing marked improvements in the TDR group in glucose regulation, modestly greater reductions in blood pressure, and a slight improvement in triglyceride levels, but no difference in cholesterol fractions (table 2). Thus, the TDR reduced overall cardiovascular risk to a greater extent than usual care (table 2).

**Table 2:** Primary, secondary, and exploratory outcomes by group allocation

Outcomes	Mean (SD) change from baseline					Adjusted difference (95% CI)	P value
	Usual care group	No	TDR group	No			
3 months:							
Weight (kg)*	−3.3 (4.2)	97	−13.3 (6.3)	114		−9.6 (−11.0 to −8.2)	<0.001
Waist circumference (cm)†	−4.6 (4.9)	94	−13.1 (7.7)	111		−8.1 (−9.9 to −6.4)	<0.001
Fat mass (kg)†	−3.0 (4.3)	95	−10.7 (6.2)	109		−7.1 (−8.6 to −5.6)	<0.001
Systolic blood pressure (mm Hg)†	3.5 (15.2)	96	−2.6 (15.8)	113		−5.8 (−9.1 to −2.4)	0.001
Diastolic blood pressure (mm Hg)†	0.5 (8.9)	96	−4.4 (9.3)	113		−3.9 (−5.9 to −1.8)	0.001
6 months:							
Weight (kg)*	−4.5 (6.2)	94	−15.1 (8.7)	108		−9.6 (−11.6 to 7.7)	<0.001
Waist circumference (cm)†	−7.0 (7.2)	89	−15.4 (9.7)	102		−8.3 (−10.6 to −6.0)	<0.001
Fat mass (kg)†	−4.8 (5.6)	86	−12.8 (9.6)	94		−7.9 (−9.9 to −5.9)	<0.001
Systolic blood pressure (mm Hg)†	4.0 (14.0	92	0.3 (16.7)	105		−3.3 (−6.9 to 0.3)	0.07
Diastolic blood pressure (mm Hg)†	0.4 (9.3)	92	−3.5 (11.4)	105		−2.8 (−5.2 to −0.4)	0.02
Quality of life:							
EQ-5D (index)†	0.03 (0.15)	73	0.07 (0.20)	92			
EQ-5D (VAS)†	7.0 (17.5)	74	15.5 (18.2)	93			
OWL-QOL†	10.6 (14.8)	74	17.4 (20.5)	92			
12 months:							
Weight (kg)‡	−3.1 (7.0)	95	−10.7 (9.6)	104		−7.2 (−9.4 to −4.9)	<0.001
No (%) lost ≥5% weight	30 (31.6)	95	76 (73.1)	104		6.5§ (3.4 to 12.2)	<0.001
No (%) lost at least ≥10% weight	14 (14.7)	95	47 (45.1)	104		4.9§ (2.4 to 9.9)	<0.001
Waist circumference (cm)†	−5.5 (7.3)	91	−10.5 (9.1)	99		−6.0 (−8.2 to −3.7)	<0.001
Fat mass (kg)*	−4.1 (6.5)	93	−10.4 (8.5)	100		−5.8 (−7.9 to −3.7)	<0.001
Systolic blood pressure (mm Hg)*	2.9 (15.2)	93	−1.6 (16.4)	100		−2.9 (−6.4 to 0.6)	0.1
Diastolic blood pressure (mm Hg)*	0.3 (9.3)	93	−4.2 (11.1)	100		−3.1 (−5.5 to −0.7)	0.01
HbA_1c_ (mmol/mol)*	−1.0 (7.7)	75	−3.2 (8.8)	91		−2.2 (−4.4 to 0.0)	0.05
Fasting glucose (mmol/L)†	0.1 (1.3)	75	−0.5 (1.8)	89		−0.4 (−0.8 to −0.1)	0.02
Fasting insulin (pmol/L)†	−10.4 (91.6)	72	−21.8 (41.8)	87		−18.0 (−32.0 to −4.0)	0.01
HOMA-IR†	−0.1 (1.5)	70	−0.5 (1.2)	86		−0.4 (−0.7 to-0.2)	0.003
HOMA-β (%)†	−15.0 (83.8)	70	−12.5 (39.7)	86		−9.8 (−22.9 to 3.4)	0.15
HOMA-S (%)†	−4.6 (70.4)	70	28.8 (47.5)	86		30.9 (16.4 to 45.5)	<0.001
Total cholesterol (mmol/L)	0.0 (0.9)	78	−0.2 (0.9)	91		−0.2 (−0.5, 0.04)	0.11
HDL cholesterol (mmol/L)†	0.1 (0.3)	78	0.2 (0.3)	91		0.1 (0.0 to 0.2)	0.09
LDL cholesterol (mmol/L)*	−0.1 (0.7)	73	−0.1 (0.6)	87		0.0 (−0.2 to 0.2)	0.8
Triglycerides (mmol/L)†	0.1 (0.6)	76	−0.3 (1.0)	89		−0.4 (−0.6 to −0.1)	0.002
QRISK2 (%)†	0.0 (2.1)	88	−0.9 (2.6)	100		−1.0 (−1.7 to −0.3)	0.01
Quality of life:							
EQ-5D (index)†	0.07 (0.14)	93	0.09 (0.17)	100			
EQ-5D (VAS)†	9.2 (17.0	96	13.0 (18.7)	101			
OWL-QOL†	14.0 (16.7)	94	17.0 (20.9)	99			

VAS=visual analogue scale; OWL-QOL=obesity and weight loss quality of life; HOMA=homeostatic model assessment; HOMA-IR=insulin resistance; HOMA-β=steady state β cell function; HOMA-S=insulin sensitivity; HDL=high density lipoprotein; LDL=low density lipoprotein.

* Secondary outcome.

† Exploratory outcome.

‡ Primary outcome.

§ Odds ratio.

Summary statistics are presented for exploratory analyses of the effect of treatment on quality of life. The TDR group showed greater improvement in EQ-5D and OWL-QOL score at six months and 12 months than the usual care group (table 2).

### Adverse events

Overall, adverse events were common and mild in both groups. Sixty nine (51%) participants in the TDR group and 41 (30%) in the usual care group experienced at least one adverse event (Fisher’s exact test: P<0.001) (see supplementary table S3), meaning that for every five people one would experience an adverse event due to the TDR. The most common adverse events where there was a greater incidence in the TDR group were constipation (1 in 7), fatigue (1 in 12), headache (1 in 17), and dizziness (1 in 22) (table 3). Most of these adverse events were mild, with only 15 (11%) in the TDR group and 17 (12%) in the usual care group classed as moderate or severe, meaning that they interfered with normal functioning (Fisher’s exact test: P=0.85) (see supplementary table S2). One participant in the TDR group experienced the serious adverse event of admission to hospital for abdominal pain as a result of diverticulitis; evidence of any causal relation with the trial intervention was lacking because the adverse event occurred after randomisation but before the participant initiated the TDR programme.

**Table 3:** Number (percentage) of participants allocated to usual care or total diet replacement (TDR) programme reporting an adverse event

Adverse events*	Usual care group (n=140)†	TDR group (n=138)†	Total
Gastrointestinal disorders:			
Abdominal discomfort	3 (2)	3 (2)	6 (2)
Upper abdominal pain	0 (0)	3 (2)	3 (1)
Breath odour	0 (0)	3 (2)	3 (1)
Constipation	0 (0)	20 (15)	20 (7)
Dry mouth	0 (0)	4 (4)	4 (1)
Nausea	0 (0)	3 (2)	3 (1)
Painful defaecation	0 (0)	4 (3)	4 (1)
General disorders:			
Asthenia	0 (0)	3 (2)	3 (1)
Fatigue	1 (1)	12 (9)	13 (5)
Influenza-like illness	4 (3)	3 (2)	7 (3)
Thirst	0 (0)	3 (2)	3 (1)
Infections:			
Lower respiratory tract infection	0 (0)	3 (2)	3 (1)
Nasopharyngitis	7 (5)	4 (3)	11 (4)
Investigations: Scan	3 (2)	0 (0)	3 (1)
Nervous system disorders:			
Dizziness	2 (1)	6 (4)	8 (3)
Headache	3 (2)	11 (8)	14 (5)
Psychiatric disorders:			
Irritability	0 (0)	3 (2)	3 (1)
Respiratory, thoracic, and mediastinal disorders:			
Oropharyngeal pain	2 (1)	2 (1)	4 (1)

* Events of any severity that occurred in more than 2% of the sample.

† Two people in the usual care group and four in the TDR group withdrew consent for use of data after randomisation.

## Discussion

Primary care referrals of people who are obese to treatment with a total diet replacement (TDR) programme in the community resulted in a weight reduction −7.2 kg (95% confidence interval −9.4 to −4.9 kg) more than usual care at one year, with statistically significantly greater improvements in glucose control, diastolic blood pressure, and triglyceride levels, but not other lipid fractions. Among participants randomised to the TDR programme, 73% lost 5% or more of their baseline body weight and 45% lost 10% or more compared with 32% and 15% of participants in the usual care group, respectively. Although adverse events were more common in the TDR group, moderate or severe events occurred at similar frequency between the two groups.

### Strengths and limitations of this study

This trial tested the effectiveness of a TDR programme for the routine treatment of people who are obese, without specific comorbidities, in a generalist care setting. We recruited patients who are typical of those seen in primary care and who were seeking support for weight loss. We found that weight loss did not vary by age, sex, socioeconomic status, or diabetes status, and together these factors suggest that this programme could be readily implemented and benefits realised across the population. In this study the intervention involved referral to a commercial provider. Previous studies have used specially trained health professionals or specialist research staff to provide the behavioural support, which is unlikely to be a scalable model for national roll-out across the NHS given current constraints on workforce. Loss to follow-up was slightly lower than in most weight loss trials.^8^ The observed difference in weight between treatment groups was greater than 5 kg in all sensitivity analyses. It is, however, limited by the relatively short duration of follow-up and the absence of direct evidence on the incidence of weight related disease or the cost effectiveness of the intervention. The formula food products are designed to be nutritionally complete, but we do not have data on nutrient intake during the programme or after the reintroduction of food, nor did we measure physical activity.

Funding for this trial was provided in large part by the provider of the intervention. This was an investigator initiated trial, however, with the idea for the trial and the protocol developed by the research team and the data analysed independently by a National Institute for Health Research accredited clinical trials unit, according to a prespecified statistical analysis plan with prior approval from the independent trial steering committee. This was a pragmatic trial where we estimated the net effect of an intervention, knowing that interventions may change other aspects of care.^20^ Although we excluded people using drugs for weight loss, other drugs can affect body weight.^21^ However, randomisation is likely to have distributed comorbidities equally by arm and therefore adjustments in drugs that could affect body weight are likely to be similar.

Our findings were obtained with one particular TDR programme and may have been different if participants were referred to another programme. However, the average weight change in this programme was similar to a study in which the diet was supported by a specially trained member of primary care staff^22^ rather than a lay counsellor, and is comparable to the average weight loss in our meta-analysis of very low energy diets, which involved a variety of products and providers.^11^ Although the population we sampled was heterogeneous for socioeconomic status, it was not as deprived as the UK population as a whole, or those people with type 2 diabetes who were enrolled in a previous trial testing a TDR programme.^22^ The subgroup analysis showed no evidence that more deprived people received less benefit; however the power to detect such interactions was limited, so there is only weak evidence to suggest the treatment effect is similar in all socioeconomic groups. The proportion of participants from non-white ethnic groups, although representative of the local population, was too small to allow any meaningful subgroup analyses. Nonetheless, our study shows that this model of referral to an external provider, which is readily available at scale, is effective, and some evidence suggests it may generalise to other programme providers and across subgroups of the population.

### Comparisons with other studies

The TDR programme comprised 12 weekly support sessions followed by three further monthly sessions together with the use of food replacement products, and it is likely that both the support and food replacement were important. In the meta-analysis of very low energy diet programmes, a very low energy (<800 kcal/day) diet proved less effective than behavioural support that aimed for modest energy restriction, whereas programmes incorporating behavioural support alongside a very low energy diet were more effective than support programmes aimed at modest energy restriction.^11^ Currently, behavioural support for weight loss in primary care is constrained by limited interest in the topic, the lack of a defined programme, and competing demands, with the result that few patients receive support for weight loss.^6^ In this pragmatic trial the nurse programme was planned to comprise support over 12 weeks. This is likely to be more than the usual care received by most people who are obese in primary care; however, it provided considerably less input than the TDR programme. The level of behavioural support in the TDR group is comparable to that provided in intensive lifestyle intervention studies such as the US diabetes prevention programme, which offered 16 sessions in the first 24 weeks. The 10.7 kg weight loss seen with TDR is greater than the 7 kg weight loss observed in the lifestyle group of the US diabetes prevention programme. Thus it appears that the weight loss observed in the TDR programme reflects both the TDR diet component and the support provided, and it is likely that either alone would be less effective than the package together.

Participants in this trial had a BMI of at least 30, and many were at risk of weight related morbidity—although at baseline only 15% had diabetes and 23% had hypertension. Evidence suggested that the intervention enhanced blood glucose control across several measures and there was a statistically significantly greater improvement in triglyceride levels although not in cholesterol fractions. The improvement in blood pressure at three months was statistically significant, but the difference was not significant thereafter for systolic blood pressure. We measured blood pressure on one occasion only, which gives an imprecise measure of the true value, and the trial was not sufficiently powered to detect the small difference that might be expected in blood pressure outcomes. Overall, the changes we observed were consistent with the magnitude of weight loss achieved.^23^ Data from other weight loss studies would suggest that this is likely to reduce the incidence of diabetes and cardiovascular disease and prevent premature mortality, commensurate with the greater reduction in 10 year cardiovascular risk seen in the TDR group.^3^ ^24^ Changes in quality of life suggested greater improvements in the TDR group than usual care group, consistent with evidence from a systematic review that greater weight loss produces improvements in quality of life.^25^ In the longer term there may be additional QALY gains as a result of a lower incidence of disease. A systematic review of dietary interventions for weight loss for adults who were obese showed a significant reduction in risk of death (risk ratio 0.82, 95% confidence intervals 0.71 to 0.95).^24^

Anecdotal reports suggest that many people are concerned that TDR programmes with severe energy restriction will be unpopular and intolerable, but our results suggest otherwise. We used a similar recruitment method to other trials and achieved a similar uptake,^8^ suggesting that people did not discriminate against this type of programme. Adverse events were more common in the TDR group but almost all mild and there was no statistically significant difference in events of moderate or greater severity. Clinicians who were initially unfamiliar with these programmes were able to adjust drugs appropriately. Together, these data suggest that TDR programmes would be effective, acceptable, and well tolerated if offered broadly. The system of referring patients to a commercial provider used in this study achieved similar absolute weight losses in the TDR groups as seen in DiRECT in people with type 2 diabetes who were from more deprived regions^22^ (DiRECT 10.0 (SD 8.0) kg, DROPLET 10.7 (SD 9.6) kg). Referral to a commercial provider is already used for other types of weight loss support and offers the potential to reduce rather than increase the workload for healthcare professionals.

In this pragmatic trial, we accepted that participants may seek to lose weight in ways other than by following their assigned programme, including purchasing additional support. Although the TDR group were offered a rescue package that permitted participants to return to the TDR stage for up to four weeks at any time during weeks 13-24 if weight was regained, none took up this offer. However, 5% of those assigned to the TDR programme did not follow the protocol and paid for additional behavioural support and formula food products to continue weight loss rather than transition to the weight loss maintenance phase. At 12 months the proportions who reported attempting to lose weight were similar in both arms, with around seven in 10 doing so. Of these, 38% in the TDR group remained in contact with a TDR consultant, presumably purchasing formula food products and receiving support, whereas only 5% of the usual care group reported continuing to follow the programme prescribed by a nurse. In addition, 6% of participants in the TDR group and 16% in the usual care group attended another commercial weight loss programme, usually a community weight loss group. Thus, these additional interventions probably contributed to overall weight loss and differences observed. This phenomenon is common to most weight loss trials; for example, at 12 months—nine months after the end of a 12 week referral to a commercial weight loss programme—19% of people initially referred to the programme were paying for continuing support, compared with 9% who were allocated to a self help arm but nevertheless sought help at their own expense.^8^ Continued use of a successful programme seems to be more common in those who achieve initial success. In a weight loss maintenance trial enrolling people who had lost at least 5% of their body weight after a 12 week NHS referral to a commercial weight loss programme, 62% continued their programme over the next three months at their own expense and 42% continued during the next nine months.^26^ This suggests that the motivation created by a successful weight loss intervention, often prompted by an NHS funded intervention, can be an important stimulus to ongoing self management, which contributes to the health outcomes observed.

### Implications of this research

Current clinical guidelines recommend that this type of diet is reserved for people in whom short term weight loss is a priority—for example, before bariatric or knee replacement surgery, and they are not recommended as routine weight loss interventions.^12^ ^13^ This presumably reflects concerns that weight loss is short lived. This trial shows that TDR leads to greater weight loss at one year than an intervention based on usual food, nine months after the TDR phase of treatment. Although, on average, some weight was regained after the programme end at 24 weeks until the final follow-up, this also occurred in the usual care group. Most weight loss programmes report average weight regain after the end of the intervention,^27^ but a proportion of participants successfully maintain clinically important weight losses. Here 45% of participants in the TDR group had lost 10% of their baseline body weight six months after the end of the intervention, compared with only 15% in the usual care group. Despite a common supposition that weight regain is related to the rate of weight loss this is not supported by experimental evidence. In a study that directly tested this hypothesis, the rate of weight regain was similar for participants who were supported to achieve 15% or more weight loss using either a very low energy diet or more moderate energy restriction over a longer duration.^28^ Since the adverse health consequences of obesity relate to the duration of excess weight as well as its magnitude,^3^ ^29^ the greater weight loss and comparable rates of average weight regain implies that the greater initial weight loss achieved with TDR programmes will be associated with greater improvements in long term health outcomes.

The NHS does not routinely offer this type of programme, and many primary care doctors are wary about supporting people who choose to use a TDR programme because they are unfamiliar with this approach or have concerns about the safety of such interventions. This trial should provide reassurance. General practitioners were given guidance to reduce, or stop, drugs for patients taking oral hypoglycaemic agents or antihypertensives at the start of the diet and to monitor these patients at four weeks. Weight loss at four weeks is a strong predictor of long term success, provides an opportunity to adjust drugs based on early weight change, and in this trial this approach did not give rise to an excess of adverse events.^30^ We included detailed elicitation of adverse events, and no unexpected or related adverse events occurred during the 12 weeks of TDR, and no cases of cholecystitis occurred during an extended reporting period to 24 weeks.

### Conclusion

A TDR programme combining nutritionally complete formula food products with behavioural support seems to be acceptable, well tolerated, and leads to greater weight loss with larger improvements in cardiovascular risk than currently available weight loss programmes offered in primary care.

**What is already known on this topic**

A systematic review of trials recommending very low energy intakes (≤800 kcal/day) showed that weight loss at one year was −10.3 kg (difference −3.9 kg, 95% confidence interval −6.7 to −1.1 kg) greater than the comparator behavioural support programmes
All the trials were conducted in specialist obesity clinics or research centres, however, and none were conducted in routine primary care
Results from the Diabetes in Remission Clinical Trial showed that a total diet replacement (TDR) programme with support provided by primary care staff for people with type 2 diabetes led to a similar weight loss, with almost half of patients in remission after one year

**What this study adds**

Compared with regular weight loss support from a practice nurse, a programme of weekly behavioural support and TDR providing 810 kcal/day (3389 kJ/day) led to substantially greater weight loss and greater improvements in the risk of cardiometabolic disease
Mean weight change at 12 months in the TDR group was −10.7 kg, a difference of −7.2 kg (95% confidence interval −9.4 to −4.9 kg) compared with usual care
Almost half (45%) of people achieved a weight loss of more than 10% at one year

## Web extra

Extra material supplied by authors

## Notes

Ethical approval: This trial was approved by South Central Oxford B NHS Research Ethics Service Committee (NHS REC No 15/SC/0337).

Data sharing: Requests for access to data from the DROPLET study should be addressed to the chief investigator at susan.jebb@phc.ox.ac.uk. All the individual participant data collected, after deidentification (including the data dictionary) will be available on request following publication. All proposals requesting data access will need to complete a data request form with details of the research question and analysis plan. All proposals will require the approval of the investigator team before any data are released.

Transparency: The guarantor (SAJ) affirms that the manuscript is an honest, accurate, and transparent account of the study bring reported; that no important aspects of the study have been omitted; and that any discrepancies from the study as planned (and, if relevant, registered) have been explained.
