# Supplementary material for: Doctor Referral of Overweight People to Low Energy total diet replacement Treatment (DROPLET): pragmatic randomised controlled trial
Source: BMJ. 2018 Sep 26;362:k3760. doi: 10.1136/bmj.k3760 (PMC6156558; doi:10.1136/bmj.k3760)
Supplement: Supplementary file 1 — Supplementary information: appendix [file astn044188.ww1.pdf]

Supplementary Appendix

Table of Contents:

Full Inclusion and Exclusion criteria	2
Medication adjustment guidelines	3
Figure S1: Treatment effect by sub-group	4
Table S1: Typical Nutritional Composition of meal replacements used in TDR	5
Table S2: Adjusted treatment effects under different missing data approaches	6
Figure S2: Pattern mixture modelling	7
Table S3: Adverse events analysis	8
Table S6: 12 month outcomes for participants with a baseline diagnosis of type 2 diabetes	9

Full list of Inclusion and Exclusion criteria

Inclusion:

Participants were included in the study if the met the following criteria:

- Participant is willing and able to give informed consent for participation in the study.
- Aged 18 years or above.
- Body Mass Index ≥30 kg/m^2.
- Likely to benefit from weight loss in the Primary Care physician's opinion.

Exclusion:

The following criteria were used to exclude individuals for whom weight loss might not be safe, those who may have difficulty adhering to TDR intervention, or those with medical conditions that were a contraindication to the TDR programme.

- Currently or recently (within 3 months of study entry) attended a weight management programme or currently participating in another weight loss study.
- Had bariatric surgery, or scheduled bariatric surgery.
- Pregnant, breastfeeding, or planning to become pregnant during the course of the study.
- Receiving insulin therapy
- Heart attack or stroke within the last 3 months
- Heart failure of grade II New York Heart Association and more severe
- Angina, arrhythmia, including atrial fibrillation or prolonged QT syndrome
- Taking MAOI medication
- Taking anticoagulant medication (e.g. warfarin)
- Taking varenicline (smoking cessation medication)
- Chronic renal failure of stage 4 or 5
- Active liver disease (except NAFLD) a past history of hepatoma or within 6 months of onset of acute hepatitis.
- People having active treatment for cancer other than skin cancer treated with curative intent by local treatment only or people taking hormonal or other long-term secondary prevention treatment after initial cancer treatment.
- Active treatment or investigation for possible or confirmed gastric or duodenal ulcer. Maintenance treatment with acid-suppression is not a contra-indication.
- Porphyria
- Scheduled for surgery within 12 months
- A member of household is already enrolled in the study
- Unwilling to provide blood samples
- Patients that the Primary Care physician judges not able to meet the demands of either treatment programme or measurement schedule. This may include severe medical problems not listed above or severe psychiatric problems including substance misuse that make following the treatment programme or adhering to the protocol unlikely.

Medication adjustment guidelines

Medication changes

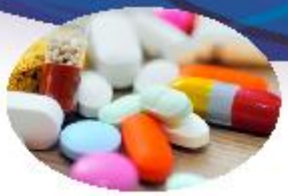

Guidance on making adjustments to your patients' medications

This guidance aims to help you make these medication adjustments, but please use your clinical judgement or contact the lead physician for this study.

TYPE 2 DIABETES	
**Patient currently takes:**	**Recommendation**
Metformin	HALF daily dose
Sulphonylurea	STOP
Glitazone	STOP
Glinide	STOP
DPP IV inhibitor	STOP
Acarbose	STOP

At the end of the weight loss phase, re-assess patients requirements for oral diabetic therapies using HbA1c measurements or a finger prick blood glucose measurement.

HYPERTENSION		
**Patient currently takes:**	**Current dose**	**Recommendation**
Loop Diuretic:		
Furosemide	≤ 40 mg daily	STOP
	80 – 120 mg daily	REDUCE by 40 mg daily
	≥ 120 mg daily	REDUCE by 40 mg daily
Bumetamide	≤ 1 mg daily	STOP
	2-3 mg daily	REDUCE to 1mg daily
	≥ 3 mg daily	REDUCE by 1mg daily
Thiazide Diuretic		STOP
β Blocker	Used for hypertension	STOP
	Other uses	CONTINUE
α Blocker		HALF daily dose
Ca channel blocker		HALF daily dose
ACE inhibitors or ARBs	Used for hypertension	STOP
	Used for heart failure	HALF daily dose

LIPID DRUGS	
**Patient currently takes:**	**Recommendation**
Fibrates	STOP
Statins	CONTINUE
Ezetimibe	CONTINUE

Figure S1: Treatment effect by sub-groups

Subgroup	N	Treatment effect [95% CI]
**Diabetes status**		
None	164	-6.60 [-9.00, -4.10]
Type 2	35	-10.20 [-15.60, -4.80]
**Sex**		
Male	86	-9.20 [-12.60, -5.80]
Female	113	-5.90 [-8.80, -2.90]
**Age group**		
19-43	54	-7.10 [-9.40, -4.80]
44-54	67	-6.90 [-10.60, -3.20]
55-78	78	-8.80 [-12.00, -4.50]
**BMI**		
BMI ≤ 35	94	-5.00 [-8.30, -1.70]
BMI >35	105	-8.70 [-11.80, -5.70]
**IMD decile***		
IMD decile ≤ 8	123	-5.90 [-9.60, -2.20]
IMD decile >8	73	-7.70 [-10.50, -4.90]
**Practice**		
Practice 1	52	-6.30 [-10.70, -1.80]
Practice 2	21	-14.00 [-20.90, -7.00]
Practice 3	36	-11.80 [-17.10, -6.50]
Practice 4	21	-2.70 [-9.70, 4.40]
Practice 5	22	-2.10 [-8.70, 4.50]
Practice 6	21	-5.80 [-12.80, 1.10]
Practice 7	15	-10.60 [-19.00, -2.30]
Practice 8	3	4.90 [-13.90, 23.70]
Practice 9	4	-3.80 [-19.10, 11.40]
Practice 10	4	-9.10 [-24.70, 6.40]
**Primary analysis**	199	-7.20 [-9.40, -4.90]

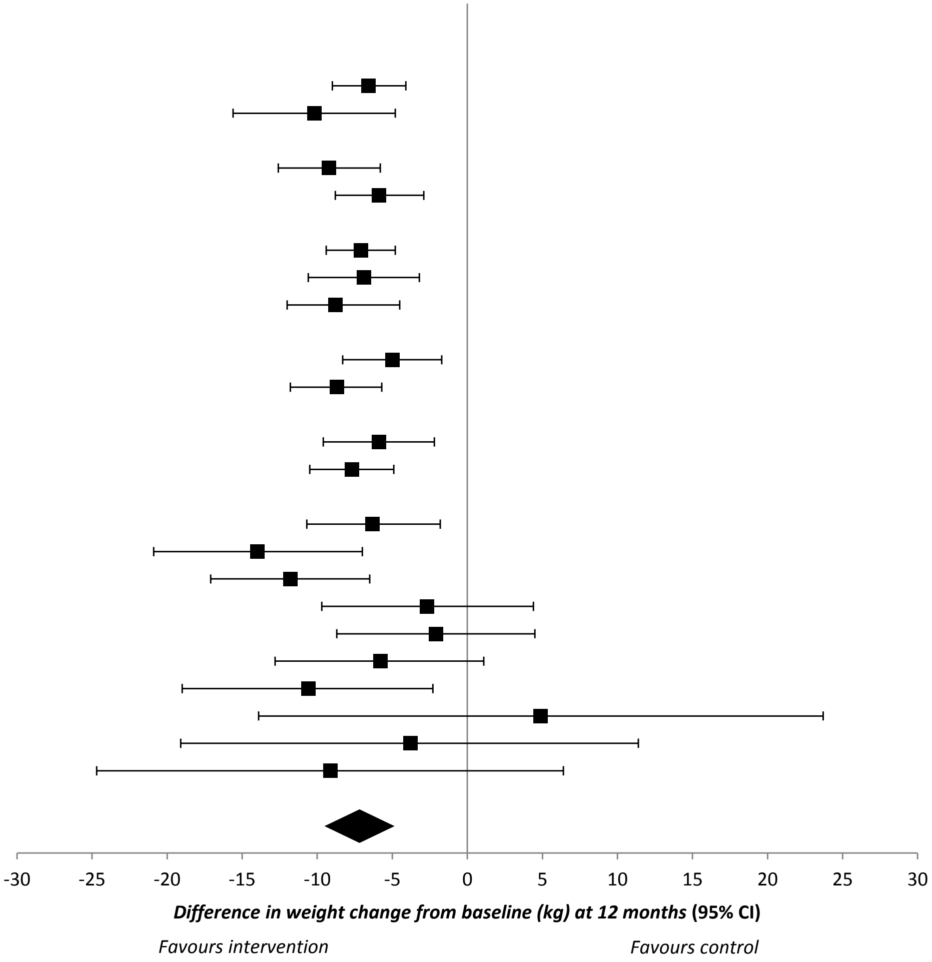

*IMD decile is an indicator of deprivation, with decile 1 being most deprived, and decile 10 the least deprived. IMD groups were compared using median split. IMD was not a pre-specified sub-group analysis, and was added after the statistical analysis plan was written, but before the primary analysis was conducted.

Table S2: Typical Nutritional Composition of meal replacement products used in the total diet replacement programme

	Per 100g	Per serving
Energy		
kJ	1565	845
kcal	370	200
Fat (g)	4.8	2.6
of which saturates (g)	0.9	0.5
Carbohydrate (g)	50.1	27.0
Of which sugars (g)	35.1	18.9
Fibre	5.2	2.8
Protein	29.2	15.8
Salt	1.2	0.6

Table S3: Adjusted treatment effects under different missing data approaches

	BOCF		LOCF		Multiple imputation		Completers only	
	Usual Care (N = 138)	TDR (N=134)	Usual care (N= 138)	TDR (N=134)	Usual Care (N=138)	TDR (N= 134)	Usual Care (N=95)	TDR (N=104)
Unadjusted weight change from baseline*	-2.1 ± 6.0	-8.3 ± 9.6	-2.7 ± 6.3	-10.2 ± 9.2	-3.5 ± 8.2	-10.2 ± 9.7	-3.1 ± 7.0	-10.7 ± 9.6
Difference between groups †	-6.1 (-8.0, -4.3)		-7.5 (-9.4, -5.6)		-6.4 (-8.5, -4.4)		-7.5 (-9.8, -5.1)	
p-value	<0.0001		<0.0001		<0.0001		<0.0001	

*Mean weight (kg) ± SD

† Adjusted mean difference (kg) (95% CI) using linear mixed effects model with fixed effects for randomisation group, baseline weight, visit and randomised group x visit interaction. Random effects accounting for practice and participant and within subject variance covariance matrix specified as unstructured. Age and sex were included as covariates as baseline values were predictive of missingness.

Figure S2: Pattern mixture modelling for weight at 12 months

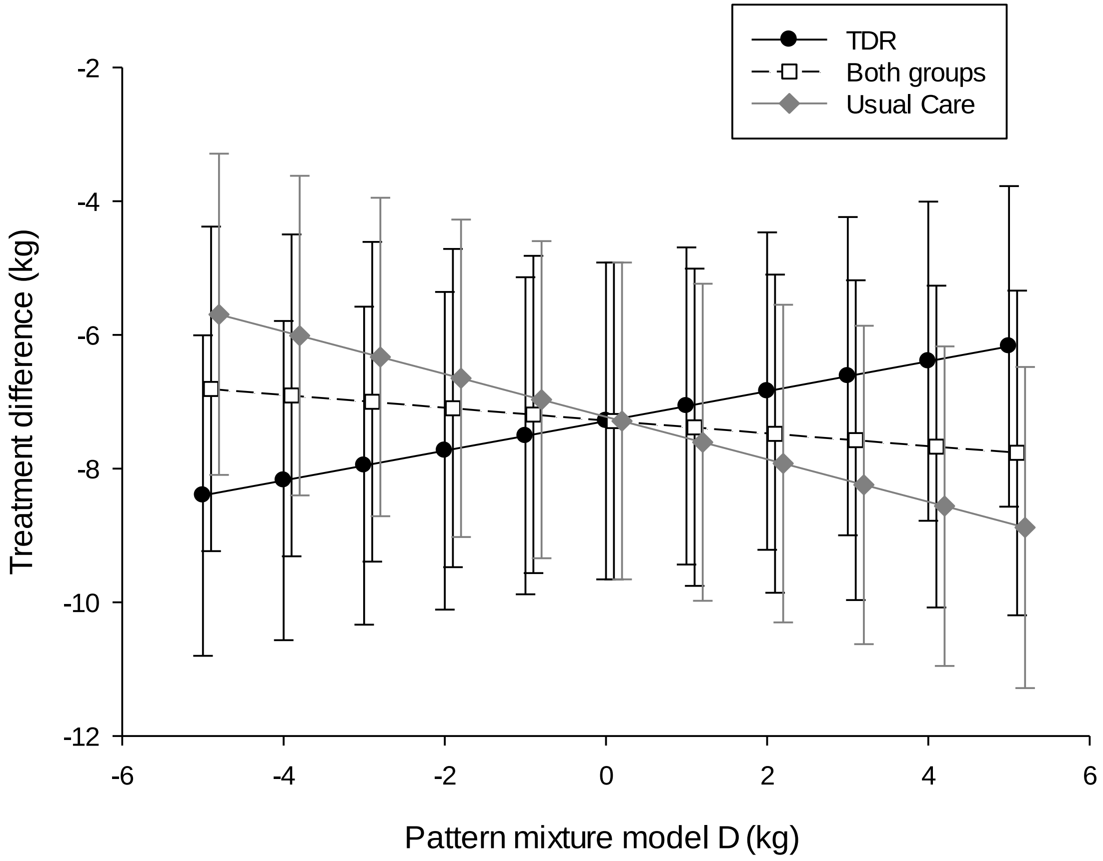

Table S5: Adverse Events analysis*

	Usual Care	Total Diet Replacement	p value
Participants reporting at least one AE n (%)	41 (29.7)	69 (51.5)	0.0003
Participants reporting a moderate or severe AE n (%)	17 (12.3)	15 (11.2)	0.85

*A logistic model would not converge, therefore a Fisher's exact test was used to test the associations between groups.

Table S6: 12 month outcomes for participants with a baseline diagnosis of type 2 diabetes

	Change from baseline (mean ± SD)				Treatment difference	
	Usual Care	n	Total Diet Replacement	n	Adjusted difference (95% CI)	p value
**12 months**						
Weight (kg)[1]	-3.2 ± 5.4	17	-13.0 ± 9.1	18	-9.9 (-14.8 ,-5.0)	<0.0001
Waist circumference (cm)[3]	-5.7 ± 6.0	17	-11.8 ± 10.9	17	-5.9 (-11.8; 0)	0.0504
Fat mass (kg)[2]	-4.3 ± 5.6	17	-13.2 ± 9.2	17	-6.3 (-12.4; -0.2)	0.0426
Systolic Blood Pressure (mmHg)[2]	5.9 ± 17.4	16	2.1 ± 19.1	17	-1.4 (-12.7; 9.9)	0.8065
Diastolic Blood Pressure (mmHg)[2]	1.0 ± 9.8	16	-2.5 ± 11.9	17	-2.9 (-9.5; -3.6)	0.3769
HbA1c (mmol/mol)[2]	-8.3 ± 15.4	17	-8.5± 17.8	17	0.09 (-13.5; 13.7)	0.9885
Fasting glucose (mmol/L)[3]	-0.3 ± 2.6	12	-1.7 ± 3.9	15	-1.0 (-2.8; -0.8)	0.2449
Fasting insulin (pmol/L)[3]	-2.2 ± 32.0	12	-26.9 ± 37.3	16	-22.7 (-46.5; 1.2)	0.0608
HOMA- IR[3]	-0.1 ± 0.6	12	-1.1 ± 2.3	15	-0.62 (-1.23;--0.01)	0.0480
HOMA β (%)[3]	-26.5 ± 100.7	12	1.2 ± 22.8	15	4.3 (-19.9; 28.5)	0.7089
HOMA S (%)[3]	5.0 ± 47.3	12	24.0 ± 29.7	15	19.4 (-13.7; 52.6)	0.3687
Total Cholesterol (mmol/L)	0.5 ± 0.8	12	0.1 ± 1.2	15	-0.37 (-1.2, 0.5)	0.1050

HDL cholesterol (mmol/L)[3]	0.1 ± 0.2	12	0.1 ± 0.5	15	-0.02 (-0.2; 0.2)	0.8047
LDL Cholesterol (mmol/L)[2]	0.2 ± 0.8	11	0.3 ± 1.0	14	-3.0 (-8.5; 2.5)	0.2327
Triglycerides (mmol/L)[3]	0.2 ± 0.9	12	-0.5 ± 1.7	15	-0.67 (-1.9; 0.6)	0.2655

[1] Primary outcome

[2] Secondary outcome

[3] Exploratory outcome
